# Life satisfaction analysis between occupational balance (OB) group and occupational imbalance (OI) group

**DOI:** 10.1371/journal.pone.0271715

**Published:** 2022-07-28

**Authors:** Yu-Jin Cha

**Affiliations:** Department of Occupational Therapy, Semyung University, Jecheon, Republic of Korea; Public Library of Science, UNITED KINGDOM

## Abstract

**Background and objectives:**

Occupation and time‐use can never be separated because they are important criteria in determining one’s lifestyle, improvements of one’s lifestyle, and even the quality of life. The purpose of this study was to identify whether there is a difference in time‐use between the occupational balance (OB) group and occupational imbalance (OI) group and to determine the factors that influence the life satisfaction of those in the OB group.

**Methods:**

This study sorted detailed activities of 9,228 participants who were over 65 years of age. Raw data of 2014 Korean Time Use Survey (KTUS) were used and the amount of time-use of older adults was classified into eight activity areas. This study classified the amount of time used by older adults for eight occupational areas, namely, activities of daily life (ADLs), instrumental ADL (IADLs), rest and sleep, education, work, play, leisure, and social participation. We identified areas of specific time differences between OB and OI groups, and confirmed variables affecting life satisfaction.

**Results:**

The analysis of time-use corresponding to the eight occupational areas showed the greatest time-use for the instrumental activities of daily living, which averaged 1513.59 minutes (56.34%). The largest effect size was social participation (d = 1.38). As a result of analyzing the factors related to the life satisfaction of the OB group, we found that those who were younger (B = 0.02, p < .001), females (B = -0.12, p < .05), had a higher level of education (B = 0.65, p < .001), had a lower need for care (B = -1.19, p < .05), had a higher income (B = -0.43, p < .001), and rural residence (B = 0.29, p < .001) tended to have a higher life satisfaction.

**Conclusions:**

This study may provide a basis for developing time‐use management and lifestyle redesign programs.

## Introduction

Most notable welfare countries and governments strive to guarantee happiness by drawing up and implementing various policies to support the goal of “improving the life satisfaction, quality of life (QoL) or well-being” with the aim of raising the level of happiness of their citizens. Subjective well-being consists of both affective well-being (i.e., positive and negative affects) and life satisfaction [1]. Following Ren, Folmer, and Arno (2018), we defined life satisfaction as a subjective assessment of the degree to which one’s needs are being met [2]. In other words, life satisfaction is a cognitive assessment of one’s own life. Life satisfaction judgments are based on their own subjective criteria, rather than necessarily reflecting outward conditions (and thus subjective labels) [3].

In most cases, the policy goal of these kinds of projects have focused on “the QoL of older adults” and that “life satisfaction” serves as a core element in attaining “QoL”. Accordingly, every policy in Korea regarding the elderly emphasizes life satisfaction as an important social task and seeks to raise it for the elderly [4]. The type of occupation and time-use can never be separated because they are important criteria in determining one’s lifestyle, improvement of life, and even life satisfaction [5]. This does not refer merely to extending one’s life, but being able to exercise control over one’s life based on being healthy at an old age.

### Occupational balance (OB) and occupational imbalance (OI) of time-use

Time-use is employed to evaluate occupational balance (OB). OB refers to a status wherein labor, rest, and the amount of time for both labor and rest are in an appropriate ratio. Occupational imbalance (OI) occurs when the excessive use of time in one area worsens one’s health or the quality of life [6, 7]. In a study by Matuska and Christiansen, OB refers to a condition in which a person can live a healthy and meaningful life in a given day-to-day occupational lifestyle [8]. Maintaining a good balance of occupation means using living time for necessary activities in an appropriate distribution. The OB perceived by an individual is likely to be influenced by the amount of time spent in everyday occupation. Time-use is assessed to evaluate the OB and occupational engagement [9].

OB is very important to human beings, and for older adults, maintaining OB adequately is connected directly to good health. This motivates humans to plan and manage their lives for a better quality of life. In particular, people who are not sufficiently provided with an opportunity to work can suffer from poor health and eventually they have difficulties with living. This demonstrates that OI may damage one’s health or cause diseases by worsening the qualitative aspects of one’s health and life [10]. Countries, therefore, need to be more aggressive in providing environments for older adults that improve their life satisfaction by maintaining the OB. In addition, countries need to establish or enforce elderly health care policies by developing health programs that consider the physical abilities of older adults or by cultivating specialists [11]. One needs training that can distribute living time appropriately as well as receive help from time intervention specialists to make this a habit.

If OI is precipitated by a time imbalance, the level of health and life satisfaction will deteriorate. Therefore, the time-use of individuals should be assessed and included in any goal setting and intervention program. OI causes an imbalance of other occupation areas as a result of a one-sided occupational performance pattern, which can ultimately worsen one’s life satisfaction and health, thus leading to impairment or disease and hindering successful aging [12]. Therefore, understanding time-use is necessary to assess the OB, which shows how to choose mandatory and non-mandatory activities according to what an individual values [9, 13].

Effective time-use shows good time management and outstanding self-management skills. These self-management skills balance a person’s needs and emotions and include proper management of temporal demands [14]. In particular, this temporal approach is suitable for investigating the daily lives of older adults, which is difficult to understand when other official and economic approaches are used [15].

Based on the results of the American Time Use Surveys (ATUS), besides rest and sleep, older adults in the US spend the greatest number of hours in leisure among the seven occupational areas. The elderly in the US spend time engaged in diverse sports activities like playing golf, walking, and swimming as well as leisure activities, including using media [16]. This was followed by IADL activities such as housework and gardening and social activities to connect with family members and friends. But they spend very little time working, playing, or learning [17].

### Occupation, participation and health promotion

An occupation is an activity that has a unique meaning and purpose in the life of an individual. It is also at the center of individual identity and competence, which influences the individual in spending time and making decisions [18]. An occupation is important enough to be considered as a part one’s life description, and experiencing meaningful occupation has a positive impact on promoting one’s health [19]. Occupations are critical to human beings, and occupations and time have an inseparable relationship with each other because people participate in some kind of occupation every hour [20].

Participation involves the balance of activities as well as diversity, meaning, and social factors of everyday use [21]. To determine the relationship between occupational engagement and health, occupational therapists have studied various concepts where OB is one of the important concepts. Experiencing meaningful occupation has a positive impact on health, and therefore, participating in a task successfully reflects the subjective value of an individual [22]. By participating in everyday occupations in a balanced matter, it is possible to promote health and improve life satisfaction [23].

### Time-use and life satisfaction

The research that focused on the relationship between physical complaints and time-use has shown that musculoskeletal discomfort is associated with the time it takes female homemakers to perform high-load repetitive tasks such as cleaning, washing utensils, and shopping [24]. Time-use and life satisfaction programs are reported to have had a positive effect on the recovery of various functions by numerous chronic and severely handicapped persons as well as by other people [13]. Kim et al. reported that they applied a lifestyle re-design program to older adults with dementia to improve their productivity as well as their life satisfaction [25].

There was a systematic review of time-use that analyzed the amount of time-use through a survey of the time spent by healthy elders. This suvey analyzed the changes in time-use by age and by year according to the occupations in the *Republic of Korea* (*ROK*) and other countries [26]. ROK is a country in East Asia located in the southern part of the Korean Peninsula. Currently, it is necessary to provide supporting data to guide health promotion and health care policies for older adults by verifying OB through assessing how they spend their day-to-day living. However, there have been few studies on the life satisfaction of older adults in which they are divided into OB and OI groups.

### The aims

The purpose of this study was to identify whether there is a difference in time-use in terms of specific criteria (eight occupational areas in total) and the factors that influence the life satisfaction of those in the OB group. To this end, we confirmed the types of use of time of older adults in a study targeting healthy older individuals over 65 years of age in the ROK by classifying them into OB and OI groups.

## Methods

### The amounts of time the participants used for occupational areas

This study identified the OB group and the OI group by using the mean and standard deviation (SD) of the quantity of time-use based on previous studies [9, 13], which reported that time-use can be used as an indicator for assessing occupational balance. This study categorized it into eight areas and the subjects of the OB group were within mean ±1 SD in all eight areas by the researcher [27]. This study selected subjects using 1 SD, a statistically stricter criterion because researchers could not control factors that might affect time-use in the investigation process and occupational balance was determined solely using time allocation. These scores were derived from the normal distribution.

### Data and sample

According to the Korea National Statistical Office (KOSTAT), the number of participants in the 2014 Korean Time Use Survey (KTUS) was 26,988 (S1 Table). The sampling frame of the 2014 KTUS was 269,664 households among the surveyed general households of the 2010 Population and Housing Census. In 2014, ROK had 652,607 citizens over the age of 65 [28]. This study sorted detailed activities of the 9,228 participants who were over the age of 65 years. Publicly available datasets were used for this study. The data used to support the findings of this study were provided by the Korean National Statistical Office (KNSO) under the license Statistics Korea (http://kostat.go.kr/portal/eng/). I obtained an exemption from the Institutional Review Board at the Semyung University (SMU-2019-03-001).

The term “healthy elders” here refers to men or women aged 65 or more who are able to walk or drive independently and manage their daily life on their own without any illness or a serious chronic disease. Samples were extracted by stratified two-stage cluster sampling. After the data were sorted according to the classification index for each region of 16 cities and provinces (i.e., first-stage extraction), 800 households were extracted using systematic selection with probability proportional to size (PPS_SYS). This study evaluated 12,000 households (800 households × 15 households = 12,000 households) using simple random sampling (SRS), which surveyed 15 households from among the initially extracted households. This study included all household members over the age of 10 (second-stage extraction). Among 27,716 household members (≥10 years old) from 11,986 households, 26,988 participants responded [29].

This study used the raw data from the 2014 KTUS of the KOSTAT and classified the amount of time used by older adults for eight occupational areas, namely, activities of daily life (ADLs), instrumental ADL (IADLs), rest and sleep, education, work, play, leisure, and social participation [20] (S2 Table). We obtained an exemption from the institutional review board approval because this study involved research of existing data, documents, records, or pathologic specimens that were publicly available or the participants were already de-identified.

ADLs are activities with a focus on taking care of one’s own body [30], which includes eating, personal hygiene, dressing, applying makeup, and so forth. IADLs are activities that support daily life within the home and community involving interactions that are more complex than ADLs; these activities include food preparation, household management, teaching children, and so forth. Rest and sleep are restorative activities that support healthy and active engagement in other occupations; these activities include sleeping, taking sick leave, idling, and so forth. Education refers to activities needed to learn and participate in an educational environment, including class time, self-study, leisure and liberal arts, learning, and so forth. Work refers to occupations with or without financial reward [31], including major jobs, agriculture, forestry and fishery work for self-consumption, and other volunteer work. Play refers to spontaneous or organized activities that provide enjoyment, entertainment, amusement, or diversion, including group games and recreation, computer and mobile games, entertainment, exploration, humor, and so on [32]. Play is another important occupation during childhood. Leisure refers to non-obligatory activities with intrinsically motivated participation during discretionary time. That is, time not committed to obligatory occupations such as work, self-care, or sleep [33] including watching TV, surfing the Internet, pursuing personal hobbies, and so on. Social participation refers to participation in the subset of activities involving social situations with others [34], including face-to-face encounters, interactions via text and mail exchanges, and other social activities.

### Statistical analysis

We conducted independent sample t-tests to investigate the time difference by area (eight areas in total) between the OB group and the OI group. We produced graphs to visually confirm the amounts of time spent on various types of activities. When Levene’s tests show that there is heteroscedasticity in the dependent variable distribution, we used the results of the Welch-Aspin test instead of the t-test. To investigate the effect of variables on life satisfaction, we performed regression analysis by applying an ordered probit model (OPM). Regression analysis has a major role in predicting the values of a dependent variable (i.e., life satisfaction) by using values from independent variables (i.e., age, gender, educational attainment, marital status, reason for needing care, economic activity status, average monthly household income, classifications of rural residence). In the 2014 KTUS of the KOSTAT survey, life satisfaction, which is a dependent variable of time-use, was measured with an ordinal-type Likert scale. The OPM is a model that depends on the error of the standard normal distribution because it does not satisfy the basic assumption of a general linear regression equation. We used the statistical programs SPSS 18.0 (SPSS Inc, Chicago, IL) and MS Excel 2010 (Microsoft Corporation, Redmond, Washington, USA) to analyze the frequency of the general characteristics of the study’s participants.

## Results

### Amounts of time-used for the various types of occupations

**Fig 1:**
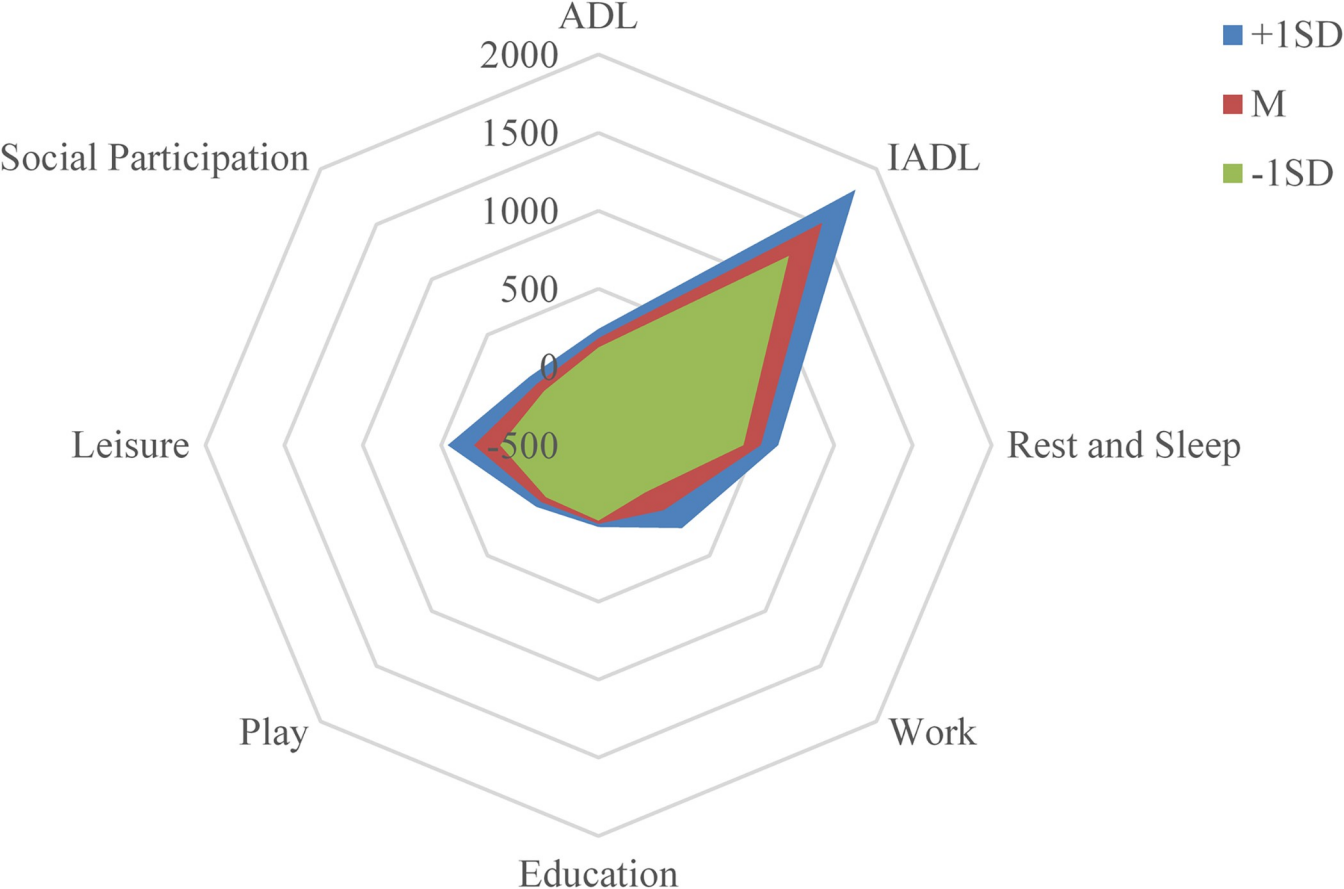
Mean value and 1 standard deviation (M ±1SD) of the time-use according to occupations.

For the amounts of time-used for the various types of occupations of the study’s participants, we obtained the following results: The greatest amount of time was used for IADL, which averaged 1,513.59 minutes (56.34%); the average time for rest and sleep was 532.82 min (19.83%), that of leisure was 292.83 min (10.9%), and that of ADL was 185.47 min (6.9%). In this study, we classified the cases within 1 standard deviation as falling in the OB group. IADL showed a range of 1213.99 to 1813.19 min, rest and sleep showed a range of 422.39 to 643.26 min, leisure time showed a range of 126.06 to 459.59 min, and ADL showed a range of 126.16 to 244.79 min. These amounts of living time are also presented visually (Fig 1 and Table 1).

**Table 1:** Time-use of occupations (min).

Occupation	-1SD	M	(%)	+1SD
ADL	126.16	185.47	(6.90)	244.79
IADL	1213.99	1513.59	(56.34%)	1813.19
Rest and Sleep	422.39	532.82	(19.83)	643.26
Work	-75.32	87.83	(3.27)	250.98
Education	-16.93	3.36	(0.13)	23.65
Play	-28.04	15.16	(0.56)	58.37
Leisure	126.06	292.83	(10.90)	459.59
Social Participation	-9.63	55.41	(2.06)	120.44

ADL: Activities of Daily Living; IAD: Instrumental Activities of Daily Living.

### Comparison of time-use between the OB group and the OI group

**Fig 2:**
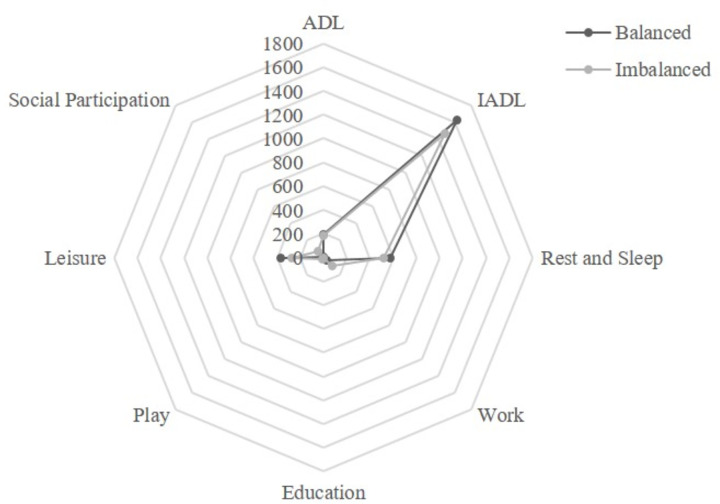
Comparison of time‐use between the occupational balance (OB) group and occupational imbalance (OI) group.

The time-use of the OB group (n = 2,153) and the OI group (n = 7,075) showed significant differences for ADL, IADL, rest and sleep, leisure, work, play, and social participation, but no significant difference for education (*p<*0.0001). Independent sample t-tests were conducted to investigate the differences in time-use for the eight occupational areas. A Welch-Aspin test was used instead of a t-test if there was heteroskedasticity of the distribution of the dependent variable as determined from the results of the Levene-test. Compared to the OI group, the OB group used more time in ADL (t = 12.39, *p<*0.0001), IADL (t = 28.21, *p<*0.0001), rest and sleep (t = -25.65, *p<*0.0001), and leisure (t = 27.99, *p<*0.0001), while it used less time in work (t = -25.65, *p<*0.0001), play (t = -5.85, *p<*0.0001), and social participation (t = -80.73, *p<*0.0001). The effect size (ES) is known as Cohen’s *d*, and the ES increases as the difference between the two groups to be compared increases. ES is a number that measures the strength of the relationship between two variables in a population or a sample-based estimate of that quantity [35]. Therefore, the occupational areas with the largest time-use difference between the two groups were social participation (1.38), followed by leisure (0.66), IADL (0.59), and work (0.51) (Fig 2 and Table 2).

**Table 2:** Comparison of time‐ use between the occupational balance group and occupational imbalance group.

Occupations	Occupational Balance (n = 2153)	Occupational Imbalance (n = 7075)	t	*p*
	M±SD	M±SD		
ADL	197.84±50.04	181.71±61.37	12.39***	*p* < 0.001
IADL	1630.77±179.43	1477.93±319.11	28.21***	*p* < 0.001
Rest and Sleep	571.42±94.62	521.08±112.22	20.66***	*p* < 0.001
Work	34.02±80.69	104.20±177.73	-25.65***	*p* < 0.001
Education	3.96±22.05	3.18±19.72	1.48	.140
Play	10.87±36.86	16.47±44.88	-5.85***	*p* < 0.001
Leisure	372.18±145.34	268.68±165.37	27.99***	*p* < 0.001
Social Participation	4.99±7.89	70.75±67.00	-80.73***	*p* < 0.001

***p < .001.

### Factors related to life satisfaction of the OB group

In order to investigate the factors related to the life satisfaction of the OB group, we conducted regression analysis by applying an ordered probit model (OPM). Life satisfaction, which is a dependent variable of KTUS, is rated on a 5-point scale ranging from ①Very Unsatisfactory to ⑤Very Satisfactory, and the order among categories was meaningful.

The static effect was significant in the case of age (B = 0.02, p < .001). The static effect was more significant in males than in females (B = -0.12, p < .05), in middle school than no educational attainment (B = 0.28, p < .01), in high school than no educational attainment (B = 0.48, p < .001), in college and university than no educational attainment (B = 0.65, p < .001), not requiring help for activity of daily life (ADL) than other reasons (B = -0.68, p < .001), not requiring help for ADL than stroke (B = -1.19, p < .01), not requiring help for ADL than disability (B = -1.00, p < .001), monthly pay over 3 million KRW compared to less than 1 million KRW (B = -0.43, p < .001), monthly pay over 3 million KRW compared to less than 2 million KRW (B = -0.32, p < .001), and lastly rural residence than non-rural residence (B = 0.29, p < .001).

The analysis showed that life satisfaction was higher in individuals who had attended middle school, high school, college, or above than in individuals of young age who were female and had no education. Regarding requiring care, it turned out that with a lower need for care for stroke, disability, and other reasons, a higher than average annual household income (higher than 3 million KRW), and rural residence, life satisfaction was higher (Table 3). Of the total 9,228 adults over 65 years old, 2,848 worked (30.9%), 6,380 did not work (69.1%), 602 had full-time jobs (6.5%), and 516 had part-time jobs (5.6%).

**Table 3:** Factors related to life satisfaction of occupational balance group.

Predictor	Category	B	SE	Wald	p
Age		0.02	0.00	0.02***	*p* < 0.001
Gender	Male	-0.12	0.05	-0.12*	.027
	Female	0.00		0.00	
Educational attainment	Elementary school	0.10	0.07	0.10	.160
	Middle school	0.28	0.09	0.28**	.001
	High school	0.48	0.09	0.48***	*p* < 0.001
	Over college and university	0.65	0.11	0.65***	*p* < 0.001
	No educational attainment	0.00		0.00	
Marital status	Married	0.06	0.06	0.06	.265
	Not married	0.00		0.00	
Reason for needing care	Dementia	-0.51	0.30	-0.51	.088
	Stroke	-1.19	0.39	-1.19**	.002
	Disability	-1.00	0.20	-1.00***	*p* < 0.001
	Other reasons	-0.68	0.13	-0.68***	*p* < 0.001
	Not need	0.00		0.00	
Economic activity status	Working	0.11	0.06	0.11	.057
	Not working	0.00		0.00	
Average monthly household income	Less than 1 million KRW	-0.43	0.07	-0.43***	*p* < 0.001
	One million KRW ~ less than 2 million KRW	-0.32	0.07	-0.32***	*p* < 0.001
	2 million KRW ~ less than 3 million KRW	-0.14	0.08	-0.14	.071
	Over 3 million KRW	0.00		0.00	
Classifications of rural residence	Rural residence	0.29	0.07	0.29***	*p* < 0.001
	Non-rural residence	0.00		0.00	
Weekday for research	Weekdays	-0.03	0.05	-0.03	.510
	Weekend	0.00		0.00	
		Model: -2 Log Likelihood = 17300.05, χ^2^ = 874.31***, p < .001.			
		Goodness-of-Fit test: χ^2^ = 21370.68***, p < .001.			
		Pseudo R^2^: Cox and Snell = .090, Nagelkerke = .098, McFadden = .036.			

***p < .001

**p < .01

*p < .05.

The KRW is the national currency of South Korea.

## Discussion

In this study, we investigated whether there are differences in time-use for eight occupational areas and which factors influence the life satisfaction of those in the OB group. To this end, we investigated the use of time for various types of activities by older adults with a focus on healthy individuals over the age of 65 in the ROK by classifying them into OB and OI groups.

The results showed that the time used for various types of occupations was the highest for IADL, followed by rest and sleep, and ADL, respectively. IADLs are activities that support daily living in the home and community, which require more complex interactions than ADLs. For example, ADLs include care, home care, shopping, religious and spiritual activities, food preparation, cleaning, driving, and community mobilization. Korean older adults were found to spend the greatest amount of time in a day on IADLs and leisure activities, which are static indoor activities. This finding agrees with those of previous studies, which found that older adults spend most of their time doing everyday household chores as IADL and some other time taking care of grandchildren or family members [17].

A comparison of time-use for ADL, IADL, rest and sleep, leisure, work, play, and social participation showed significant differences between the OB and OI groups, whereas the time-use for education showed no significant difference. The factor showing the largest ES was social participation, followed by leisure, IADL, and work. Thus, the occupational areas with the largest time-use difference between the two groups were social participation, followed by leisure, IADL, and work.

The OB group spent greater amounts of time than the OI group on leisure, IADL, rest and sleep, and ADL, while they spent lower amounts of time than the OI group on work, play, and social participation. This aligns with the findings of previous studies, which reported that older adults who enjoy continued leisure activities show a lower tendency towards depression and a higher satisfaction with everyday life than those who do not participate in leisure activities, regardless of their type [36].

Compared to the OI group, the OB group spent more time in ADL, IADL, rest and sleep, and leisure and less time in occupational, play, and social participation. This result contradicts the results of a study referring to social participation and proper occupation at an old age, which are related to mutual exchanges with other people as essential elements for improving and maintaining the life satisfaction of older adults [37]. According to socioemotional selectivity theory (SST), the aged consciously reduce the frequency of social contact to spend more time on the emotionally compensating relationship, such as family or friends. Therefore, the aged belonging to the OB group are found to seek to enhance life satisfaction by reducing social networks selectively. Based on SST, this study proposes that in developing social relationship activity services for the older adults’ life satisfaction, they minimize emotional risk and maximize positive emotional experience by spending more time with close friends or family members, rather than forming new relationships.

An analysis of the factors related to the life satisfaction of the OB group found the following. A higher life satisfaction corresponded to being younger and female as well as having higher levels of education, a lower need for care, higher income, and rural residence. This is consistent with the findings of Jeon, who discovered that younger, female participants had a greater tendency to use their time in an occupational balance. The study results also showed that younger adults were more active, and that females showed higher levels of ADL and IADL in comparison to males [10].

These results are consistent with the findings of studies that the higher the level of education was, the higher the life satisfaction of older adults was, in line with the expectation that those with higher education have better financial status and higher life satisfaction than others [38]. In other words, the higher the economic and educational levels of older adults are, the more important and decisive the close relationship with self-development and socio-cultural activities is to the life satisfaction of older adults [37]. The findings of this study show that those with a higher average monthly household income have a higher daily life satisfaction, despite the insignificance of economic activity status.

We can estimate the hours of living in the type of occupational balance with the amount of the time used through an analysis of the data of the 2014 Time Use Survey of the KOSTAT. Many studies on occupational balance have reported that it has an important influence on human life satisfaction and health [6, 9, 12].

This study had limitations in investigating the life satisfaction and the importance of time-use in KTUS as well as in understanding the use of living time according to the social environment and the characteristics of individuals. We expect that future surveys of living time may overcome these limitations. Moreover, because sociocultural differences among countries have some effect on the ways that older adults spend their time, future studies should analyze the factors that determine OB based on theories of sociocultural differences among nations in terms of time-use. Although this study was based on a survey of healthy older adults, the lack of a measure of health was a limitation. In previous studies that reviewed the influence of one’s health condition on life satisfaction, the subjective satisfaction with one’s health was an important variable that predicted life satisfaction [39]. In future studies, variables should be applied to measure both subjective and objective health conditions.

This study has significance in that it analyzed the living time of older adults over 65 years of age in terms of occupational type. This analysis will also help clients that have difficulty in time-use as well as healthy elders perform their daily tasks of occupational performance. Various methods should be suggested to maintain the OB for the successful aging of older adults.

## Conclusion

Through the results of this study, we gained insights into the relationships among occupation, old age, health, and life satisfaction, so that we can effectively establish old-age preparation plans for current older adults as well as future generations. We expect that the results will have important implications and could be used as a basis for use in mediation and program development. This study could guide the development of time-use management and redesign lifestyle programs for older adults to practice independent disease prevention and health maintenance.

## Supporting information

S1 Table Demographic characteristics of the research participants. (DOCX)

S2 Table Reclassification of eight activity areas based on detailed activities in KTUS 2014. (DOCX)
