# Correction: Life satisfaction analysis between occupational balance (OB) group and occupational imbalance (OI) group

**DOI:** 10.1371/journal.pone.0314576

**Published:** 2024-11-21

**Authors:** Yu-Jin Cha

In the Data and sample subsection of Methods, there is an error in the fifth sentence of the first paragraph. The correct sentence is: I obtained an exemption from the Institutional Review Board at the Semyung University (SMU-2017-EX-03-003).
