# Supplementary material for: Life satisfaction analysis between occupational balance (OB) group and occupational imbalance (OI) group
Source: PLoS One. 2022 Jul 28;17(7):e0271715. doi: 10.1371/journal.pone.0271715 (PMC9344896; doi:10.1371/journal.pone.0271715)
Supplement: S1 Table — (DOCX) [file pone.0271715.s001.docx]

**S1 Table.** **Demographic characteristics in the research participants**.

| **Items** | **Classifications** | **N(%)** |
| --- | --- | --- |
|  |  |  |
| Age | younger elderly  (65~74 years) | 5,436 (58.91) |
|  | older elderly  (over 75 years) | 3,792 (41.09) |
| Gender | Male | 3,800 (41.18) |
|  | Female | 5,428 (58.82) |
| Educational attainment | No educational attainment | 1,900 (20.59) |
|  | Elementary school | 3,498 (37.91) |
|  | Middle school | 1,500 (16.25) |
|  | High school | 1,520 (16.47) |
|  | Over college and university | 810 (8.78) |
| Marital status | Not married | 3,604 (28.11) |
|  | Married | 5,624 (43.87) |
| Reason for needing care | Not need | 8,654 (93.78) |
|  | Dementia | 62 (0.67) |
|  | Stroke | 40 (0.43) |
|  | Disability | 102 (1.11) |
|  | Other reasons | 370 (4.01) |
| Economic activity status | Working | 2,87 (31.19) |
|  | Not working | 6,350 (68.81) |
| Average monthly household income | Less than 1 million KRW | 7,080 (76.70) |
|  | One million KRW ~ less than 2 million KRW | 1,420 (15.40) |
|  | 2 million KRW ~ less than 3 million KRW | 422 (4.60) |
|  | Over 3 million KRW | 306 (3.30) |
| Classifications of agricultural residence | Agricultural residence | 1,694 (18.36) |
|  | Non-agricultural residence | 7,534 (81.64) |
| Size of residential area ^*^ | County/Eup | 2,774 (30.06) |
|  | Urban/Dong | 6,454 (69.94) |
| Weekday for research | Weekdays | 5,464 (59.21) |
|  | Weekend | 3,764 (40.79) |
| Whether to have co-habitants | One person | 2,330 (25.25) |
|  | Having co-habitants | 6,898 (74.75) |
