# Supplementary material for: Life satisfaction analysis between occupational balance (OB) group and occupational imbalance (OI) group
Source: PLoS One. 2022 Jul 28;17(7):e0271715. doi: 10.1371/journal.pone.0271715 (PMC9344896; doi:10.1371/journal.pone.0271715)
Supplement: S2 Table — (DOCX) [file pone.0271715.s002.docx]

**S2 Table.** **Reclassification of eight activity areas on detailed activities in KTUS 2014.**

| **Contents of activities** | **Code number** | **Activity areas** |
| --- | --- | --- |
| Sleeping | A120 | R |
| Insomnia | A140 | R |
| Sick leave | A340 | R |
| Idling | G970 | R |
| Eating | A220 | A |
| Snacking and drinking | A240 | A |
| Personal hygiene | A920 | A |
| Dressing, make-up | A940 | A |
| Other personal care activity | A990 | A |
| Class time | C120 | E |
| Breaks between classes | C140 | E |
| Self-study at school | C160 | E |
| School/University events | C180 | E |
| Other School/University activities | C190 | E |
| Taking courses at private institutions | C220 | E |
| Taking broadcasting/online courses | C240 | E |
| Self-study | C260 | E |
| Other education than school activities | C290 | E |
| Leisure and liberal arts learning | G940 | E |
| Self-therapy | A320 | I |
| Getting medical care services | A360 | I |
| Getting beauty services | A960 | I |
| Food preparation | D120 | I |
| Making snacks or desserts | D140 | I |
| Clearing table, washing dishes | D160 | I |
| Getting food-related services | D180 | I |
| Laundry | D220 | I |
| Fixing and producing homeware and shoes | D240 | I |
| Getting homeware and shoes related services | D280 | I |
| Cleaning | D320 | I |
| Tidying up home | D340 | I |
| Dumping trash | D360 | I |
| Household management | D420 | I |
| Household item management and production | D440 | I |
| Getting residence related service | D460 | I |
| Getting household item related services | D480 | I |
| Vehicle maintenance | D520 | I |
| Getting vehicle maintenance services | D540 | I |
| Pet care | D620 | I |
| Plant care | D640 | I |
| Getting pet and plant care services | D660 | I |
| Off-line shopping | D720 | I |
| On-line shopping | D740 | I |
| Offline purchase of service | D760 | I |
| Online purchase of service | D780 | I |
| Other shopping related behavior | D790 | I |
| Organizing housekeeping book | D920 | I |
| Using the services of financial institutions | D940 | I |
| Using the services of public office etc. | D960 | I |
| Other home maintenance | D990 | I |
| Physical Care (under 10 years old) | E120 | I |
| Teaching children (under 10 years old) | E140 | I |
| Reading books /Playing with children (under 10 years old) | E160 | I |
| Nursing children (under 10 only) | E180 | I |
| Other cares of children (under 10 only) | E190 | I |
| Physical Care (over 10 years old) | E220 | I |
| Studying (over 10 years old) | E240 | I |
| Nursing (over 10 years old) | E260 | I |
| Other care (over 10 years old) | E290 | I |
| Nursing (spouse) | E320 | I |
| Other care (spouse) | E390 | I |
| Nursing (parents and grandparents living together) | E420 | I |
| Other caring (parents and grandparents living together) | E490 | I |
| Nursing (other family members or roommate) | E520 | I |
| Other caring (other family members or roommate) | E590 | I |
| Nursing (parents and grandparents not living together) | E620 | I |
| Other caring (parents and grandparents not living together) | E690 | I |
| Nursing (other family members not living together) | E720 | I |
| Other caring (other family members not living together) | E790 | I |
| Helping housekeeping activity | F360 | I |
| Religious activities | G320 | I |
| Participating in religious gatherings | G340 | I |
| Other religious activities | G390 | I |
| Traveling due to personal care | H120 | I |
| Commuting | H220 | I |
| Traveling due to other works | H240 | I |
| Traveling due to learning | H320 | I |
| Traveling due to home maintenance | H420 | I |
| Traveling related to caring family members living together | H520 | I |
| Traveling related to caring family members not living together | H540 | I |
| Traveling related to volunteer/participation | H720 | I |
| Traveling related to fellowship and leisure activities | H820 | I |
| Others relevant to traveling | H920 | I |
| Reading books | G210 | L |
| Reading newspapers | G220 | L |
| Reading magazines | G230 | L |
| Watching TV | G240 | L |
| Watching Videos etc. | G250 | L |
| Listening to the radio | G260 | L |
| Listening to records/CDs/Tapes/MP3 | G270 | L |
| Internet surfing | G280 | L |
| Other leisure activities related to media | G290 | L |
| Smoking | G960 | L |
| Other leisure activities | G990 | L |
| Personal hobbies | G930 | L |
| Movie theatre / Video room | G410 | L |
| Concert/theatre | G420 | L |
| Visiting museum/exhibition halls | G430 | L |
| Watching sports games | G440 | L |
| Tourism / Driving tour | G450 | L |
| Other cultural and tourism activities | G490 | L |
| Walking | G510 | L |
| Running/ Jogging | G520 | L |
| Climbing | G530 | L |
| Bicycle / In-line skate | G540 | L |
| Personal exercise | G550 | L |
| Ball game | G560 | L |
| Fishing / Hunting | G570 | L |
| Other sports / Leports | G590 | L |
| Mass game / plays | G910 | P |
| Computer / mobile games | G920 | P |
| Entertainment | G950 | P |
| Mandatory participation | F120 | S |
| Other participation | F190 | S |
| Caring for your acquaintances | F320 | S |
| Helping income-generating activities | F340 | S |
| Other helping | F390 | S |
| Face-to-face encounter | G120 | S |
| Interactions via video and voice exchange | G140 | S |
| Interactions via text and mail exchange | G160 | S |
| Other fellowship activities | G190 | S |
| Ceremonies such as wedding, funeral etc. | G620 | S |
| Major job | B110 | W |
| Side jobs | B120 | W |
| Unpaid agriculture, forestry and fishery work on family farm | B130 | W |
| Unpaid work on family farm besides agriculture, forestry and fishery | B140 | W |
| Agriculture, forestry and fishery work for self-consumption | B150 | W |
| Break at workplace | B170 | W |
| Work related training | B180 | W |
| Other job-related activities | B190 | W |
| Job seeking | B220 | W |
| Participation related to children's education | F140 | W |
| Volunteering for national and local events | F220 | W |
| Activities related to the neglected class | F240 | W |
| Activities related to people in disaster | F260 | W |
| Other volunteering work | F290 | W |

*A* activities of daily living, *I* instrumental activities of daily living, *R* rest and sleep, *W* work, *E* education, *P* play, *L* leisure, *S* social participation
